# Global Adult Tobacco Survey Data as a Tool to Monitor the WHO Framework Convention on Tobacco Control (WHO FCTC) Implementation: The Brazilian Case

**DOI:** 10.3390/ijerph9072520

**Published:** 2012-07-23

**Authors:** Liz Almeida, André Szklo, Mariana Sampaio, Mirian Souza, Luís Felipe Martins, Moysés Szklo, Deborah Malta, Roberta Caixeta

**Affiliations:** 1 Instituto Nacional de Câncer José Alencar Gomes da Silva, Rua Marquês de Pombal, 127, 7° andar, Centro, CEP 20230-240, Rio de Janeiro, RJ, Brasil; Email: aszklo@inca.gov.br (A.S.); miriancs@inca.gov.br (M.S.); lfmartins@inca.gov.br (L.F.M.); mszklo@jhsph.edu (M.S.); 2 Secretaria de Estado de Saúde do Rio de Janeiro, Rua Graça Aranha, 182/6º. andar, CEP 20030-001, Rio de Janeiro, RJ, Brasil; Email: m.autran@gmail.com; 3 Secretaria de Vigilância em Saúde, Ministério da Saúde, Esplanada dos Ministérios, Bloco G, CEP 70058-900, Brasília, DF, Brasil; Email: deborah.malta@saude.gov.br; 4 Pan American Health Organization, 525 23rd Street NW, Washington, DC 20037, USA; Email: caixetro@paho.org

**Keywords:** tobacco, smoking, epidemiology, health promotion, health policy

## Abstract

The Global Adult Tobacco Survey (GATS) was conducted in Brazil to provide data on tobacco use in order to monitor the WHO FCTC implementation in the country. It was carried out in 2008 using an international standardized methodology. The instrument included questions about tobacco use prevalence, cessation, secondhand smoke, knowledge, attitudes, media and advertising. Weighted analysis was used to obtain estimates. A total of 39,425 interviews were conducted. The prevalence of current tobacco use was 17.5%, (22.0%, men; 13.3%, women). The majority of users were smokers (17.2%) and their percentage was higher in rural areas (20.4%) than in urban areas (16.6%). About 20% of individuals reported having been exposed to tobacco smoke in public places. Over 70% of respondents said they had noticed anti-smoking information in several media and around 65% of smokers said they had considered quitting because of warning labels. About 30% of respondents had noticed cigarette advertising at selling points and 96% recognized tobacco use as a risk factor for serious diseases. Data in this report can be used as baseline for evaluation of new tobacco control approaches in Brazil, vis-à-vis WHO FCTC demand reduction measures.

## 1. Introduction

The World Health Organization Framework Convention on Tobacco Control (WHO FCTC) was developed as a response to the globalization of the tobacco epidemic [1]. This treaty establishes a set of targets and activities to be implemented by the parties to the convention. It aims to reduce both tobacco production and tobacco consumption.

Ever since the late 1980s, when the National Tobacco Control Program (NTCP) was created, a broad set of legislative, health care, educational and economic measures have been implemented (Table 1) [2]. In 2003, the National Committee for the WHO FCTC Ratification was created by Presidential Decree, with 16 Ministries and Secretariats (Health, Education, Agriculture, Environment, Justice, Industry and Commerce, Communication, Science and Technology, Labor, Treasure, Planning and Budget, Agriculture Development, National Secretariat Anti-Drugs, National Security, Special Secretariat for Women Policy and Presidential Cabinet). In 2005, the WHO FCTC was ratified in Brazil. Since then, the Committee was renamed as the National Committee for the WHO FCTC Implementation to ensure the tobacco control actions continue to be supported through a multi sectorial approach. At present, the WHO FCTC is the National Tobacco Control Policy—a legal mandate.

Brazil’s participation in the Global Adult Tobacco Survey (GATS) promoted by the World Health Organization was consistent with the need to develop mechanisms to assess the impact of these actions, as nationwide data on tobacco use are scarce. It was also in accordance with Article 20 of the WHO FCTC encouraging the establishment of programs for national, regional and global surveillance of the magnitude, patterns, determinants and consequences of tobacco consumption [1].

The GATS-Brazil was carried out by the Brazilian Geographic and Statistics Institute with the cooperation of the Ministry of Health. It had two objectives: to support national policies for tobacco control and to enable international comparability of its results. Other international institutions also contributed to the survey, including the United States Centers for Disease Control and Prevention, the World Health Organization, the Pan American Health Organization and the Johns Hopkins Bloomberg School of Public Health. 

In order to provide useful information for devising strategies to prevent initiation, to promote cessation and to protect people against secondhand smoke, the aim of the present study was to evaluate the demographic correlates of tobacco products use, cessation attempts, exposure to secondhand smoke, access to awareness campaigns about the risks of smoking and people’s perception of these risks among individuals aged 15 years-old and older.

**Table 1:** Tobacco control legislation in Brazil between 1986 and 2008.

Year of implementation	Legislative action	Description
**1986**	Population awareness	Creation of the National No-Tobacco Day that have been celebrated, every year, on 29 August.
**1988**	Bans/Restrictions on advertising and sponsorship of tobacco products	Determination that the advertising of tobacco will be subject to legal restrictions and will bear warning about the dangers of smoking.
**1996**	Health warnings	Insertion of text-warning messages on the packages and on the marketing material of tobacco products.
**1996**	Protection against the risks of exposure to secondhand tobacco smoke pollution	Prohibition of the use of any smoked tobacco product in public or private collective facilities. However, allowance of smoking in designated smoking areas, properly insulated and ventilated conveniently.
**1998**	Restriction of the access to tobacco products	Determination that the advertisement of cigarettes in the country, including its sale display, be available only on packets, bags or other types of receptacle containing twenty units of cigarettes.
**1998**	Protection to young people	Prohibition of the sale, supply or delivery, to children or adolescents (under 18), of products whose components may cause physical or psychological dependence.
**1999**	Control and inspection of tobacco products	Creation of Brazilian Health Surveillance Agency which also regulate, control and inspect tobacco products.
**2000**	Protection to young people	Prohibition of children and adolescents from participation in the advertisement of tobacco products.
**2000**	Bans/Restrictions on advertising and sponsorship of tobacco products	Restriction of the advertising of tobacco products to the display of posters, billboards and posters inside sales outlets, banning it, therefore, in magazines, newspapers, television, radio and billboards. Prohibition of advertising by electronic media, including the Internet, the advertising indirectly contracted, also called merchandising and advertising in stadiums, tracks, stages or similar sites. Prohibition of sponsorship of national sporting events and cultural activities.
**2000**	Restriction of the access to tobacco products	Prohibition of the sale by mail, the distribution of samples or tokens and the marketing of tobacco products in educational and health care institutions.
**2001**	Health warnings	Determination that advertising materials and packages of tobacco products, except those destined for exportation, include warnings with pictures and also the National Quitline toll-free number.
**2001**	Treatment and support to smokers	Creation of the nationwide toll-free telephone cessation counseling service (Quitline).
**2001**	Control and inspection of tobacco products	Prohibition of the use, on packages or advertising material, of descriptors, such as classes, ultra-low yields, low yields, mild, light, soft, smooth, moderate yields, high yields, and others.
**2002**	Protection against the risks of exposure to secondhand tobacco smoke pollution	Prohibition of the use of tobacco products in aircraft and other vehicles of collective transportation.
**2002**	Protection to young people	Prohibition of the production, importation, marketing, advertising and distribution of food in the same form of cigarettes, cigars, cigarillos, or any other tobacco product
**2003**	Health warnings	Insertion of warnings with pictures on the packages and on the marketing material of tobacco products
**2003**	Framework Convention on Tobacco Control	Creation of the National Commission for WHO FCTC Ratification in Brazil.
**2003**	Restriction of the access to tobacco products	Prohibition of the sale of tobacco products on the Internet.
**2004**	Treatment and support to smokers	Implementation of universal and free access to cognitive-behavioral and pharmacological treatment of smokers in the network of primary care and medium complexity of the Brazilian Health System.
**2005**	Framework Convention on Tobacco Control	Ratification of the WHO FCTC by the Congress
**2006**	Tax on tobacco products	Increase in excise taxes. Overall, the tax rate is 60.0% of the retail price.
**2007**	Control and inspection of tobacco products	Cigarette manufacturers are obliged to install production metering equipment, allowing the control and tracking of the products and enabling the legitimate identification of the product’s origin, repressing the illegal production and importation, as well as the marketing of counterfeits.

## 2. Methodology

### 2.1. Design, Sampling, Instruments and Data Collection

Individuals aged 15 years or older were included in the survey. Data collection was conducted in households. The sampling plan employed was the same as that used by the annual National Household Sampling Survey (NHSS), with an extra stage to randomly select one resident aged 15 or older from each household [3].

The NHSS sampling plan consisted of a probabilistic household sample obtained through three selection stages: primary units (municipalities); secondary units (census tracts); and tertiary units (households, including private households and housing units in collective households). The GATS sample size of households was 1/3 of that of the NHSS sample size of households. Considering a non-response rate of 20% (which included empty and destroyed households, refusals and incomplete interviews), and to secure interviews from 40,000 individuals, a sampling size of 50,000 was aimed at representing approximately one household for every three in the NHSS sample. This sample size was estimated with the objective of generating estimates for the country as a whole and for five geographical regions, stratified by gender and residence (urban and rural) for the GATS-Brazil.

The questionnaire included information about the household and selected individual characteristics (socio-demographic factors, tobacco consumed, smokeless tobacco, cessation, secondhand exposure to tobacco smoke, media exposure and knowledge). The questionnaires were developed by a committee of international experts and revised by the Brazilian component of the survey’s executive committee, so as to adapt it to the country’s reality and needs [2].

For the GATS-Brazil data collection, Personal Digital Assistants (PDA) were used. In each of Brazil’s 27 states, a regional team was responsible for data collection and decentralized evaluation of the survey data. Each team had a regional coordinator, supervisors, interviewers, and administrative and informatics support.

### 2.2. Statistical Analysis

For the GATS-Brazil expansion factors, the selection of one resident 15 years of age or older in the fourth stage was also considered, as well as a correction for when this resident did not answer a given question. Additionally, the GATS-Brazil weighting factors were adjusted so that the population estimates by gender would correspond to the NHSS total population by gender estimated from the whole sample, in each of the geographical regions.

For data analysis, Stata 9.0 [4] was used as the “survey” module to provide data analysis of epidemiological surveys with complex sampling strategies. Proportions estimates and their respective 95% confidence interval were inspected by sex and place of residence. For all comparative analyses, F-test (Fisher-Snedocor) was used to assess statistical significance.

### 2.3. Ethical Aspects

The Brazilian Geographic and Statistics Institute (IBGE) complies with the norms proposed by the United Nations Statistics Commission, abiding by the principles of impartiality, equal access, maintenance of professional and ethical standards, responsibility and transparency, prevention against data misuse, efficiency and confidentiality. For this reason, the IBGE is exempt from submitting all its survey proposals to the National Ethics Committee and it does not have to obtain Informed Consent from the individuals interviewed.

## 3. Results

The survey was carried out in 2008, from October to December. The overall response rate was 95.2%. A total of 39,425 interviews were conducted, 33,680 in urban areas (85.4%) and 5,745 in rural areas (14.6%). The expanded sample corresponded to about 146 million individuals aged 15 years and older. The sample included a higher proportion of women than men (54.2% *versus* 45.8%).

### 3.1. Tobacco Use Prevalence

The prevalence of smoked and smokeless tobacco use in Brazil was estimated to be 17.5% in 2008 (22.0% among men and 13.3% among women). This proportion corresponded to around 25 million individuals. The majority of the tobacco users were smokers (17.2%). The prevalence rates in men and women were, respectively, 21.6% and 13.1%. Among current smokers, most used tobacco products daily. The percentage of current smokers was higher in rural areas (20.3%) than in urban areas (16.6%) (*p* value < 0.001) (Table 2). Nevertheless, due to the high concentration of the population in urban areas (82%), the absolute number of smokers was considerably higher in these areas.

**Table 2:** Percentage of adults ≥15 years old, by place of residence, gender and smoking status, GATS Brazil, 2008.

Gender and smoking status				Brazil		Place of residence			
						Urban		Rural	
				%	[95% Confidence Interval]	%	[95% Confidence Interval]	%	[95% Confidence Interval]
**Overall**									
	Current tobacco smoker ^1^			17.2	[16.7–17.7]	16.6	[16.1–17.1]	20.3	[19.1–21.7]
		Daily smoker		15.1	[14.6–15.5]	14.5	[14.0–15.0]	18.0	[16.8–19.3]
		Occasional Smoker		2.1	[1.9–2.3]	2.1	[1.9–2.3]	2.3	[1.9–2.9]
			Occasional Smoker, formerly daily	0.9	[0.8–1.0]	0.9	[0.8–1.0]	0.9	[0.7–1.2]
			Occasional Smoker, never daily	1.2	[1.1–1.4]	1.2	[1.1–1.3]	1.4	[1.1–1.9]
	Non-smoker			82.8	[82.3–83.3]	83.4	[82.9–83.9]	79.6	[78.3–80.9]
		Former daily smoker		14.1	[13.7–14.5]	13.9	[13.5–14.4]	15.0	[13.9–16.1]
		Never daily smoker		68.7	[68.1–69.3]	69.5	[68.8–70.1]	64.7	[63.1–66.2]
		Former occasional smoker		4.1	[3.8–4.3]	4.1	[3.9–4.4]	3.9	[3.3–4.6]
		Never smoker		64.7	[64.0–65.3]	65.3	[64.7–66.0]	60.8	[59.1–62.5]
**Male**									
	Current tobacco smoker ^1^			21.6	[20.8–22.3]	20.6	[19.8–21.4]	26.3	[24.4–28.3]
	Daily smoker			18.9	[18.2–19.6]	18.0	[17.2–18.8]	23.3	[21.5–25.2]
		Occasional Smoker		2.7	[2.4–2.9]	2.6	[2.3–2.9]	3.0	[2.4–3.9]
			Occasional Smoker, formerly daily	1.0	[0.9–1.2]	1.0	[0.8–1.2]	1.1	[0.7–1.6]
			Occasional Smoker, never daily	1.6	[1.4–1.9]	1.6	[1.4–1.8]	1.9	[1.4–2.7]
	Non-smoker			78.4	[77.6–79.2]	79.4	[78.6–80.2]	73.7	[71.7–75.6]
		Former daily smoker		17.2	[16.6–17.9]	17.1	[16.4–17.9]	17.8	[16.3–19.3]
		Never daily smoker		61.2	[60.3–62.1]	62.3	[61.3–63.2]	55.9	[53.8–57.9]
		Former occasional smoker		4.2	[3.8–4.5]	4.3	[3.9–4.7]	3.6	[2.9–4.5]
		Never smoker		57.0	[56.1–57.9]	58.0	[57.0–59.0]	52.2	[50.1–54.4]
**Female**									
	Current tobacco smoker ^1^			13.1	[12.6–13.7]	13.1	[12.5–13.7]	13.5	[11.9–15.1]
		Daily smoker		11.5	[11.0–12.1]	11.5	[10.9–12.0]	11.9	[10.5–13.5]
		Occasional Smoker		1.6	[1.4–1.8]	1.6	[1.4–1.8]	1.5	[1.1–2.2]
			Occasional Smoker, formerly daily	0.7	[0.6–0.9]	0.7	[0.6–0.9]	0.7	[0.4–1.2]
			Occasional Smoker, never daily	0.8	[0.7–1.0]	0.8	[0.7–1.0]	0.9	[0.6–1.3]
	Non-smoker			86.9	[86.3–87.4]	86.9	[86.3–87.5]	86.5	[84.8–88.1]
		Former daily smoker		11.2	[10.7–11.7]	11.1	[10.6–11.7]	11.7	[10.2–13.4]
		Never daily smoker		75.7	[74.9–76.4]	75.8	[75.0–76.6]	74.8	[72.6–76.9]
		Former occasional smoker		4.0	[3.7–4.4]	4.0	[3.7–4.3]	4.2	[3.2–5.4]
		Never smoker		71.7	[70.9–72.4]	71.8	[71.0–72.6]	70.7	[68.4–72.8]

Note: ^1^ Current smoker includes both daily and occasional (less than daily) smoker.

The proportion of former daily smokers was 14.1%, representing 20.1 million individuals aged 15 or older in the total population. The proportion of former daily smokers was higher among men than among women (*p* value < 0.001) (Table 2).

As shown in Table 3, most current smokers consumed cigarettes. The prevalence of use of manufactured cigarettes was 14.4% and the prevalence of the use of hand-rolled cigarettes was 5.1%. In rural, compared with urban areas, a higher proportion of straw cigarette or hand-rolled cigarette smokers was observed (13.8% *versus* 3.6%; *p* value < 0.001). The percentage of smokers of other tobacco products, such as cigars, pipes, cigarillos, Indian cigars and narguille, was low: 0.8% on average, 0.9% among men and 0.7% among women. It can also be noted (data not shown) that cigarette smoking use reflects the Brazilian social inequality, *i.e*., individuals with no or less than a year of schooling and with the lowest purchasing power have the highest smoking prevalence (*p* value < 0.001).

**Table 3:** Percentage of current tobacco users ≥15 years old, by place of residence, gender and tobacco product, GATS Brazil 2008.

Gender and tobacco product				Brazil		Place of residence			
						Urban		Rural	
				%	[95% Confidence Interval]	%	[95% Confidence Interval]	%	[95% Confidence Interval]
**Overall**									
	Any smoked tobacco product			17.2	[16.7–17.6]	16.6	[16.1–17.1]	20.4	[19.1–21.7]
		Any cigarette ^1^		17.1	[16.6–17.6]	16.5	[16.0–17.1]	20.1	[18.8–21.4]
			Manufactured cigarette	14.4	[14.0–14.9]	14.9	[14.4–15.4]	11.9	[10.9–13.0]
			Hand-rolled	5.1	[4.8–5.5]	3.6	[3.3–3.9]	13.8	[12.6–15.1]
	Other smoked tobacco ^2^			0.8	[0.7–0.9]	0.7	[0.6–0.9]	1.1	[0.8–1.6]
	Current users of smokeless tobacco			0.4	[0.4–0.5]	0.3	[0.2–0.4]	1.2	[0.9–1.6]
**Male**									
	Any smoked tobacco product			21.6	[20.8–22.3]	20.6	[19.8–21.4]	26.3	[24.4–28.3]
		Any cigarette ^1^		21.5	[20.7–22.3]	20.5	[19.7–21.4]	0.3	[24.3–28.2]
			Manufactured cigarette	17.8	[17.1–18.6]	18.3	[17.5–19.1]	15.6	[14.0–17.3]
			Hand-rolled	7.4	[6.8–7.9]	5.1	[4.6–5.5]	18.6	[16.8–20.5]
	Other smoked tobacco ^2^			0.9	[0.7–1.0]	0.8	[0.7–1.0]	1.0	[0.6–1.6]
	Current users of smokeless tobacco			0.6	[0.5–0.7]	0.4	[0.3–0.5]	1.5	[1.0–2.2]
**Female**									
	Any smoked tobacco product			13.1	[12.6–13.7]	13.1	[12.5–13.7]	13.5	[11.9–15.1]
		Any cigarette ^1^		13.0	[12.5–13.6]	13.0	[12.4–13.6]	13.1	[11.6–14.7]
			Manufactured cigarette	11.3	[10.8–11.8]	11.8	[11.3–12.4]	7.7	[6.6–9.0]
			Hand-rolled	3.1	[2.7–3.4]	2.2	[2.0–2.5]	8.3	[7.0–9.7]
	Other smoked tobacco ^2^			0.7	[0.6–0.9]	0.6	[0.5–0.8]	1.3	[0.9–2.0]
	Current users of smokeless tobacco			0.3	[0.2–0.4]	0.2	[0.1–0.3]	0.8	[0.5–1.5]

Notes:^ 1^ Includes manufactured cigarettes, hand rolled cigarettes, and kreteks; ^2^ Includes bidis, pipes, cigars or cigarillos, narguille, and other products.

For the first time in Brazil, it was possible to estimate the proportion of users of smokeless tobacco, such as snuff and chewing tobacco (Table 3). The percentage of individuals who consumed this type of product was 0.4% overall (0.6% among men and 0.3% among women), which corresponded to 420,000 men and 200,000 women. The highest prevalence of smokeless tobacco users was also found in rural areas: 1.2% *versus* 0.3% in urban areas (*p* value < 0.001).

### 3.2. Cessation

In Brazil, the quit rate (number of former smokers over the total number of ever smokers) was around 50% (Table 4). Out of the total number of individuals 15 years of age or older who smoked, 45.6% had attempted to quit smoking in the previous 12 months. Women attempted to quit smoking more frequently than men (49.5% *versus* 43.0%) (*p* value < 0.001). Around 60% of the population who smoked or had quit smoking for less than 12 months had gone to a medical doctor or health professional for some reason. Out of this total, 71% were asked whether they were smokers and 57.1% were advised to quit smoking (Table 4).

**Table 4:** Smoking cessation indicators for adults ≥15 years old, by place of residence and gender, GATS Brazil, 2008.

Smoking cessation indicators		Brazil		Place of residence			
				Urban		Rural	
		%	[95% Confidence Interval]	%	[95% Confidence Interval]	%	[95% Confidence Interval]
**Overall**							
	Smoking cessation quit rate ^1^	51.4	[50.4–52.4]	52.1	[50.9–53.2]	48.1	[45.6–50.5]
	Adults ≥15 years old who made a quit attempt in the 12 months before the interview date ^2^	45.6	[44.2–47.0]	46.1	[44.6–47.6]	43.4	[40.4–46.5]
	Adults who reported recollection of being asked if they smoked by a doctor or health care provider ^3^	71.0	[69.3–72.6]	71.5	[69.7–73.3]	67.8	[63.5–71.8]
	Adults who reported recollection of being advised to quit smoking by a doctor or health care provider ^3^	57.1	[55.3–58.9]	57.3	[55.4–59.2]	55.8	[51.3–60.2]
**Male**							
	Smoking cessation quit rate ^1^	49.8	[48.4–51.2]	50.9	[49.4–52.5]	44.9	[41.7–48.1]
	Adults ≥15 years old who made a quit attempt in the 12 months before the interview date ^2^	43.0	[41.2–44.8]	43.7	[41.7–45.8]	40.4	[36.7–44.1]
	Adults who reported recollection of being asked if they smoked by a doctor or health care provider ^3^	70.2	[67.6–72.6]	70.7	[68.0–73.4]	67.7	[61.9–73.0]
	Adults who reported recollection of being advised to quit smoking by a doctor or health care provider ^3^	55.7	[53.0–58.3]	55.9	[53.0–58.7]	54.9	[48.8–60.8]
**Female**							
	Smoking cessation quit rate ^1^	53.7	[52.2–55.2]	53.6	[52.0–55.3]	54.1	[49.9–58.3]
	Adults ≥15 years old who made a quit attempt in the 12 months before the interview date ^2^	49.5	[47.4–51.6]	49.4	[47.1–51.6]	50.4	[44.9–55.8]
	Adults who reported recollection of being asked if they smoked by a doctor or health care provider ^3^	71.8	[69.5–74.0]	72.3	[69.9–74.6]	68.0	[61.0–74.2]
	Adults who reported recollection of being advised to quit smoking by a doctor or health care provider ^3^	58.5	[56.1–60.9]	58.7	[56.1–61.2]	57.2	[50.5–63.7]

Notes: ^1^ Former smokers/ever smokers; ^2^ Among the population who smoked or had quit in the 12 months before the interview date; ^3^ Among the population who smoked or had quit in the 12 months before the interview date and have visited a doctor or health care professional.

### 3.3. Exposure to Secondhand Smoke (SHS)

Exposure to secondhand smoke at home at least once a month was reported by 27.9% of respondents (40 million individuals in Brazil, 24 million being non-smokers) (Table 5). A higher proportion reported being exposed to secondhand smoke at home in rural than in urban area, irrespective of gender (*p* values < 0.001).

**Table 5:** Percentage of adults ≥15 years old who are exposed to tobacco smoke at work, in health care facilities, in restaurants, in public transportation an in government buildings or offices, by place of residence and gender, GATS Brazil, 2008.

Adults who were exposed to tobacco smoke...	Brazil		Place of residence				
			Urban			Rural	
	%	[95% Confidence Interval]	%		[95% Confidence Interval]	%	[95% Confidence Interval]
**Overall**							
…at home at least once a month	27.9	[27.2–28.7]	26.0		[25.3–26.8]	38.7	[36.6–40.7]
... at work ^1^	24.4	[23.4–25.4]	24.3		[23.2–25.3]	26.3	[22.4–30.6]
... in public places ^2,3^	18.2	[17.6–18.8]	19.7		[19.1–20.4]	9.3	[8.3–10.5]
**Male**							
…at home at least once a month	28.9	[27.9–29.9]	26.5	[25.6–27.5]		40.6	[37.9–43.3]
... at work ^1^	28.5	[26.9–30.0]	28.3	[26.8–29.9]		31.2	[25.1–38.0]
... in public places ^2,3^	17.8	[17.1–18.6]	19.7	[18.9–20.5]		8.8	[7.5–10.2]
**Female**							
…at home at least once a month	27.0	[26.1–27.9]	25.6	[24.7–26.4]		36.4	[36.9–38.9]
... at work ^1^	20.4	[19.2–21.7]	20.3	[19.0–21.6]		22.8	[17.9–28.6]
... in public places ^2,3^	18.5	[17.8–19.3]	19.8	[19.0–20.6]		10.0	[8.5–11.7]

Notes: ^1^ In the 30 days before the interview. Among those respondents who work outside of the home and usually work indoors or both indoors and outdoors; ^2^ In the 30 days before the interview; ^3^ It includes health care facilities, restaurants, public transportation, and government buildings.

Exposure to secondhand smoke in the workplace in Brazil was reported by 24.4% of respondents who worked in enclosed places, representing around 12 million individuals in Brazil; of these, nine million were not smokers (Table 5). Women had a lower rate of secondhand smoke exposure at work than men (*p* value < 0.001). No differences were found between rural and urban areas pertaining to secondhand smoke at work.

Not including the workplace, about one in every five individuals surveyed reported exposure to tobacco smoke in public places, corresponding to 26 million individuals in Brazil, with 22 million being non-smokers. If exposure to tobacco smoke at work is also included, a total of 28 million of non-smokers were exposed to secondhand smoke. Men and women reported similar proportions of exposure to tobacco smoke in public places, such as health care facilities, restaurants, public transportation, and government buildings. Individuals living in rural areas were less often exposed to tobacco smoke (*p* value < 0.001). 

### 3.4. Media

Over 70% of all those surveyed said they had noticed anti-cigarette smoking information in different media, including television and radio, newspapers and magazines and others. Among these, the highest proportion was associated with television, even when stratified by gender (Table 6) and tobacco use. Lower percentages of exposure to tobacco information were seen in rural than in urban areas (*p* value < 0.001), but no gender differences were observed.

The percentage of smokers who reported noticing health warnings on cigarette packages was 87.7% (not shown in table). It was lower in the rural than in urban areas (*p* value < 0.001). Around 65% of people interviewed said they had considered quitting smoking because of warning labels on the cigarette packages. The percentage was higher among women compared to men (Table 6) (*p* value = 0.009). About 30% of both smokers and non-smokers had noticed cigarette advertising at points of sale. The results also showed that men and residents of urban areas reported the highest awareness of tobacco advertising in all forms (Table 6) (*p* value < 0.001).

**Table 6:** Percentage of adults ≥15 years old who noticed anti-cigarette smoking information and cigarette advertising during the 30 days before the interview date and, by place of residence and gender, GATS Brazil, 2008.

Adults who...			Brazil		Place of residence			
					Urban		Rural	
			%	[95% Confidence Interval]	%	[95% Confidence Interval]	%	[95% Confidence Interval]
**Overall**								
	... noticed anti-cigarette smoking information							
		overall	73.1	[72.2–74.0]	74.9	[74.1–75.7]	63.2	[60.3–65.9]
		on TV	63.9	[63.1–64.8]	65.5	[64.6–66.4]	55.0	[52.3–57.7]
		on the radio	30.3	[29.4–31.2]	30.0	[29.1–30.9]	31.7	[29.4–34.1]
		... thought about quitting smoking due to pictures or warning labels on cigarette packages	65.0	[63.4–66.5]	67.0	[65.4–68.6]	55.7	[51.8–59.5]
	... noticed cigarette advertising							
		overall	38.0	[37.1–38.9]	40.4	[39.5–41.3]	24.6	[22.7–26.7]
		in sales points	30.4	[29.5–31.3]	32.2	[31.3–33.2]	20.1	[18.3–22.0]
	... noticed cigarette promotion		3.4	[3.2–3.7]	3.6	[3.4–4.0]	2.1	[1.7–2.7]
**Male**								
	... noticed anti-cigarette smoking information							
		overall	72.5	[71.5–73.6]	74.8	[73.7–75.8]	61.6	[58.3–64.8]
		on TV	63.6	[62.5–64.7]	65.6	[64.5–66.7]	54.0	[50.8–57.1]
		on the radio	30.6	[29.5–31.7]	30.4	[29.3–31.6]	31.4	[28.5–34.3]
		... thought about quitting smoking due to pictures or warning labels on cigarette packages	63.5	[61.6–65.4]	65.6	[63.5–67.7]	55.4	[51.1–59.7]
	... noticed cigarette advertising							
		overall	41.8	[40.6–43.0]	44.8	[43.5–46.0]	26.9	[24.5–29.5]
		in sales points	33.9	[32.8–35.1]	36.3	[35.0–37.5]	22.5	[20.2–25.0]
	... noticed cigarette promotion		4.1	[3.7–4.5]	4.4	[4.0–4.9]	2.3	[1.8–3.1]
**Female**								
	... noticed anti-cigarette smoking information							
		overall	73.6	[72.7–74.6]	75.0	[74.0–75.9]	64.9	[61.7–68.0]
		on TV	64.2	[63.2–65.2]	65.4	[64.4–66.5]	56.2	[53.0–59.4]
		on the radio	30.0	[29.0–31.0]	29.6	[28.6–30.7]	32.1	[29.6–34.8]
		... thought about quitting smoking due to pictures or warning labels on cigarette packages	67.2	[65.0–69.3]	69.0	[66.6–71.2]	56.3	[50.0–62.3]
	... noticed cigarette advertising							
		overall	34.5	[33.5–35.6]	36.5	[35.4–37.6]	22.0	[19.7–24.6]
		in sales points	27.1	[26.2–28.1]	28.7	[27.7–29.7]	17.2	[15.1–19.5]
	... noticed cigarette promotion		2.8	[2.5–3.1]	3.0	[2.6–3.3]	1.9	[1.3–2.6]

### 3.5. Knowledge, Attitudes and Perceptions about Tobacco Use

Of the total number of respondents 15 years of age or older, 96.1% said they believed smoking could cause serious diseases. The percentages were 93% among smokers and 96.7% among non-smokers. The most frequently expressed perception was tobacco as a risk factor for lung cancer: 94.7% of the total number of people interviewed (Table 7). Over 90% of the surveyed population recognized the association between exposure to secondhand smoke and serious diseases. Awareness of the health risks related to smoking was more common in the urban areas and in smokers (*p* value < 0.001).

**Table 7:** Percentage of adults ≥15 years old who believed that smoking/breathing other people’s smoke causes serious illness, by place of residence and gender, GATS Brazil, 2008.

Adults who...			Brazil		Place of residence			
					Urban		Rural	
			%	[95% Confidence Interval]	%	[95% Confidence Interval]	%	[95% Confidence Interval]
**Overall**								
	...believed that smoking could cause							
		serious illnesses	96.1	[95.7–96.4]	96.4	[96.1–96.7]	94.2	[93.2–95.2]
		stroke	73.1	[72.3–73.9]	73.9	[73.1–74.6]	68.6	[66.2–70.9]
		heart attack	85.6	[85.1–86.2]	86.4	[85.8–87.0]	81.3	[79.5–83.1]
		lung cancer	94.7	[94.3–95.1]	95.2	[94.8–95.5]	92.1	[91.0–93.1]
	...believed that breathing other people’s smoke could cause serious illnesses in non-smokers		91.4	[90.9–91.8]	92.1	[91.6–92.5]	87.4	[85.7–88.9]
**Male**								
	... believed that smoking could cause							
		serious illnesses	95.9	[95.4–96.3]	96.2	[95.8–96.6]	94.0	[92.7–95.1]
		stroke	74.3	[73.3–75.2]	75.3	[74.3–76.2]	69.4	[66.6–72.0]
		heart attack	85.7	[84.9–86.4]	86.6	[85.8–87.4]	81.0	[78.7–83.2]
		lung cancer	94.5	[94.0–94.9]	95.0	[94.5–95.4]	92.0	[90.5–93.2]
	... believed that breathing other people’s smoke could cause serious illnesses in non-smokers		90.8	[90.2–91.4]	91.6	[90.9–92.1]	86.9	[85.1–88.6]
**Female**								
	... believed that smoking could cause							
		serious illnesses	96.3	[95.9–96.7]	96.6	[96.2–97.0]	94.5	[93.1–95.6]
		stroke	72.0	[71.1–73.0]	72.7	[71.8–73.6]	67.8	[64.8–70.6]
		heart attack	85.6	[84.9–86.3]	86.2	[85.5–86.9]	81.7	[79.5–83.7]
		lung cancer	94.9	[94.5–95.4]	95.3	[94.9–95.8]	92.3	[90.7–93.6]
	... believed that breathing other people’s smoke could cause serious illnesses in non-smokers		91.9	[91.3–92.5]	92.5	[92.0–93.1]	87.9	[85.7–89.7]

## 4. Discussion

### 4.1. Tobacco Use Prevalence

Currently, there are around 1.2 billion smokers worldwide [5]. In Brazil, in 1989, a survey covering both urban and rural areas estimated the smoking prevalence at 34.8% (43.3% among men and 27.0% among women), which corresponded to around 28 million aged 18 or older [6]. In 2003, another population-based survey [7], with the same coverage as that of the 1989 study, observed a smoking prevalence of 21.9% among individuals 18 years or older (27.1% among men and 18.4% among women). At the time, this represented around 25 million smokers. The GATS-Brazil survey showed an 18.1% (22.9% among men and 13.9% among women) cigarette consumption prevalence among individuals 18 years of age or older, reflecting an estimated total of 24 million smokers (data not shown). Most of these were daily manufactured cigarette smokers, but the most popular tobacco product in the rural area was hand-rolled cigarette.

These surveys were both cross-sectional population-based and used stratified and weighted probabilistic samples to represent the Brazilian population. As a whole, they point to a gradual decrease in the country’s smoking prevalence for both male and female [8]. Findings on gender are consistent with the postulates of Lopez *et al.*’s model applied to Brazil [9]. According to these authors, the tobacco epidemic transition is described in stages, *i.e*., tobacco use begins first among men; the subsequent dissemination of knowledge about its harms would avoid the expansion of the epidemic among women. Under this assumption, the peak smoking prevalence for women will be lower than that observed for men. Moreover, these results reinforce the importance of establishing progressively a national system for the epidemiological surveillance of tobacco consumption and related social, economic and health indicators, as stated in article 20 of the FCTC [1]. Surveillance allows countries to continue to make progress in tobacco control through the development of new strategies to prevent initiation and encourage cessation, targeted to different smoking groups [1,2]. Accordingly, it is worth emphasizing that GATS-Brazil is part of the Brazilian National System of Epidemiological Surveillance, along with household surveys conducted every five years, telephone surveys conducted yearly since 2006, and school surveys conducted periodically [10,11].

### 4.2. Cessation

The survey revealed that a significant percentage of smokers had tried to quit smoking in the previous 12 months. Women attempted to quit smoking more frequently than men, suggesting that women are more concerned about their health than men. Indeed, studies indicate that, despite living longer than men, women suffer more morbidity and psychological problems and make use of health services more frequently than men [12,13,14,15]. Another possible explanation is that women are also more concerned about daily life tobacco-specific problems such as the smell of their hair or the smell of their clothes, or are even more responsive when their children ask them not to smoke [16].

The interventions to promote smoking cessation, in course in Brazil include national cessation campaigns, dissemination of self-help materials, and health warnings in tobacco products’ packages and telephone cessation counseling (Quitlines). Additionally, the Brazilian Unified Health System started offering a tobacco dependence treatment (behavioral counseling and pharmacotherapy) in the whole country in 2004. These measures are fully consistent with article 14 of the FCTC, which encourages the development of effective measures to promote cessation of tobacco use and provision of adequate treatment for tobacco dependence, including increasing access and affordability [1].

Still, GATS-Brazil data suggest that raising awareness of tobacco-related problems among smokers and increasing accessibility to support may be not sufficient. Indeed, out of the total number of smokers who had visited a doctor in the last 12 months, only 57.1% received smoking cessation advice or help. It is, therefore, important to encourage smoking treatment in the health services network. Health professionals’ capacity and knowledge of the importance of integrating smoking cessation in their health care practices should be fostered. 

### 4.3. Secondhand Tobacco Smoke

Exposure to smoke from tobacco products is an important public policy issue [17]. The frequency of this exposure reflects the multidimensional relationship between the socio-cultural context, income, and access to places of exposure.

An alarming finding was the number of non-smokers exposed to secondhand tobacco smoke in at least one of the environments surveyed: around 22 million people. The high percentage of exposure to secondhand smoke causes much concern because it indicates that the legislation in place that time was not able to protect people from secondhand smoke exposure (Federal Law number 9294). This law has prohibited smoking in enclosed collective spaces since 1996, allowing smoking in smoking areas, *i.e*., areas assigned specially to tobacco that are properly insulated and ventilated conveniently. The work environment potentially provides up to eight hours of daily exposure to workers. As a consequence, secondhand smoke is now considered an occupational hazard [17]. 

The data obtained in the GATS survey regarding exposure to tobacco smoke underscored the urgent need for full implementation, in Brazil, of the recommendations contained in the WHO FCTC, specifically in its 8th article (protection against secondhand smoke) [1]. These data were launched in several press-conferences before a vote to modify the current legislation and its regulatory decrees, in order not to accept designated smoking areas in enclosed public spaces and workplaces. It is worth mentioning that because of flaws in the Federal Law number 9294, states and municipalities have passed series of legislative acts to fully protect the population from the secondhand smoke exposure, since this survey was conducted and before this federal law was modified in 2011. 

Thus, GATS data likely contributed to increase protection against secondhand smoke exposure in the country. For instance, a study aimed at quantifying levels of air nicotine in bars in São Paulo state, Brazil, before and after the state law that completely banned smoking in indoor places, found a 72% reduction in air nicotine in the surveyed establishments [18].

### 4.4. Knowledge and Media

The survey responses regarding knowledge and the media indicated that the country is on the right path concerning dissemination of information about the dangers resulting from the use of, and exposure to tobacco smoke. The Brazilian society has considerable knowledge of the health problems related to smoking and an acute awareness of anti-cigarette smoking information as disseminated in the general media and on cigarette packages, resulting from countless actions of the National Tobacco Control Program in the last decades and also the strong support from the media.

GATS Brazil results showed that men and residents of urban areas reported the highest awareness of tobacco advertising in all forms. Such findings may reflect heterogeneity in access to information and its relationship to individual risk perception. The differences in awareness of either anti-cigarette information or cigarette marketing by subgroups of the population suggest the need to vary communication formats by the National Tobacco Control Program so as to counter more efficiently the effects of marketing and/or promotion of tobacco products to target audiences.

The survey showed that 30.4% of smokers and non-smokers had noticed cigarette advertising at points of sale, indicating that the tobacco industry has been efficiently using these places to expose its products.

Although Brazil had legislation that restricted advertising to inside points of sale, the new law, approved in December, 2011, bans all tobacco products advertising in any place of the country. The measure will reinforce the postulates of articles 11 and 13 of the WHO FCTC, which recognizes that a comprehensive ban on advertising, promotion and sponsorship would reduce the consumption of tobacco products [1]. In Canada, for instance, cigarettes are stored out of consumers’ direct sight, following the recommendations of the WHO FCTC.

Survey informants indicated high levels of knowledge about the harms caused by smoking. A similar result was obtained in the International Tobacco Control (ITC-Brazil) survey [19], conducted in 2009 which seems to indicate that the educational interventions developed in Brazil, whether continuous or restricted, have been fulfilling their purpose. For instance, an important strategy proposed by article 11 of the WHO FCTC, has been the inclusion of health warnings on packages of tobacco products [1]. Messages were initiated in 1988 and illustrations were added in 2001. Brazil is already in the 3rd cycle of warning images on cigarette packages [20], which are very strong and have a greater impact than the previous ones. The new law that obliges health warnings to cover both sides of the tobacco product package and that stimulates the increase of the size of health warnings at points of sale is encouraged by the postulates of article 12 of the WHO FCTC [1]. In this article, it is stated that education and information should be offered together with legislation because they reinforce each other. The implementation of legislation is a tool to educate people, to reduce social stimulus for smoking and to change social acceptance. 

One of the advantages to conduct a survey using an international standardized protocol is the possibility of assessing cross-country comparisons. New papers exploring the cultural and socioeconomic differences among countries, as well as their different stages of the WHO FCTC implementation, are also important For instance, at present, only two other Latin American countries have GATS data available: Mexico and Uruguay [21]. Mexico showed the lowest tobacco use point prevalence (15.9%) and Uruguay, on the other hand, had the highest (25.0%). In terms of frequency, the majority of users were daily smokers in Brazil (88%) and in Uruguay (82%), but not in Mexico (48%). In all the countries, more than 95% of the people believe smoking causes serious illness and more than 90% believe that second-hand smoke causes serious illness.

Our findings should be interpreted cautiously, as data are subject to biases resulting from self-reporting data on smoking and secondhand smoke without biological marker confirmation, as well as recall bias. Moreover, the cross-sectional data presented do not allow us to establish a clear temporal link between the GATS data/results and their use in the assessment and formulation of tobacco control policy and practice in Brazil. 

## 5. Conclusions

The role of the GATS-as a tool to monitor for the WHO FCTC implementation-should be highlighted. It was the first national-level comprehensive survey of tobacco use in Brazil, including a broad set of legislative, health care, education and economic aspects. Its effective implementation was based on a close relationship between policymakers and the technicians who conducted the fieldwork. The survey gave information for the country as a whole, which allows the exploration of heterogeneities among the regions. It is important to select priorities and to generate other studies in the country. 

Developing an effective comprehensive tobacco control program requires careful monitoring and evaluation of existing programs and the likely development of new preventive strategies. The GATS core questions were added to the new National Health Survey, scheduled to be conducted every five years, starting from 2013. Accordingly, they will be used as baseline data for future evaluations of new tobacco control approaches to be implemented toward achieving the goals of the WHO FCTC, as well as the goals of reducing smoking prevalence that have been defined in the Strategic Plan to Tackle of Noncommunicable Chronic Diseases (NCDs) in Brazil, 2011–2022 [22]. 
